# A child with cPAN as only manifestation of FMF

**DOI:** 10.1186/1546-0096-12-S1-P361

**Published:** 2014-09-17

**Authors:** Gayane Khloyan, Gayane Amaryan, Traudel Saurenmann

**Affiliations:** 1 General Pediatrics, Arabcir MC -Institute of child and Adolescent Health, Yerevan, Armenia; 2 National Pediatric Centre for Familial Mediterranean Fever , Arabkir MC- Institute of Child and Adolescent Health, Yerevan, Armenia; 3 Canton Hospital , Winterthur, Switzerland

## Introduction

Cutaneous Polyarteritis Nodosa (cPAN) is a rare type of vasculitis affecting small-to-medium-size arteries. It is distinct from systemic PAN in that it lacks significant internal organ involvement. Familial Mediterranean Fever (FMF) is the most common inh**e**rited autoinfl**a**mmatory disease, characterized by recurrent, self–limited attacks of fever and aseptic polyser**o**sitis. PAN is considered one of the four types of vasculitis associated with FMF. FMF is widespread in Armenia and there is a higher than expected frequency of FMF-associated vasculitis which may in some cases be the first indication for MEFV mutation analysis.

## Objectives

We want to introduce the importance of MEFV gene analysis in the patients with vasculitis in population with high prevalence of FMF.

## Methods

A case report of a patient diagnosed and followed in multidisciplinary unit.

## Results

A 7 years old girl was admitted to the hospital with the following complaints: high grade fever for 3 days, stomatitis, ulcers on the feet, swelling and tenderness of the left ankle joint. Past history was unremarkable. The physical examination revealed livedo reticularis on the lower extremities, painful subcutaneous nodules and ulcers on the left leg and foot, arthritis of the left ankle joint. Blood pressure was normal. No signs of internal organ involvement were found. Initial laboratory findings indicated: mild anemia, (Hbg-100g/l), thrombocytosis -700.000/mcl. elevated acute phase reactants - leukocytes -26,000/mcl, with 81% of neutrophils, ESR 70 mm/h, and CRP 96 mg/l. An extensive diagnostic work up including blood culture and serology (ANA, anti dsDNA. ANCA, ACA, HBs Ag …), biochemical examination, bone marrow aspirates and imaging studies did not reveal any pathology. Despite of absence of FMF symptoms, genetic analysis of *MEFV* mutations was done and 2 mutations in compound heterozygous (F148Q/P369S) state were found. Along with prednisone, azathioprine, treatment with colchicine was started. Later AZA was discontinued because of intolerance. Prednisone was tapered and stopped within 6 months. Currently the patient is only on colchicine treatment and in complete remission for both diseases.

## Conclusion

In populations with a high prevalence of FMF, screening of *MEFV* mutation is recommended for patients with different types of vasculitis, even in the absence of FMF symptoms.

## Disclosure of interest

None declared

